# Necroptotic signaling orchestrates glioblastoma malignancy and potentiates temozolomide response

**DOI:** 10.1038/s41419-025-08377-3

**Published:** 2025-12-22

**Authors:** Yuanyuan Li, Yuxin Qiu, Wenqing Gao, Lu Geng, Zerui Wu, Teng Zhang, Jiasong Pan, Jianglong Lu, Danfeng Zhang, Adi Idris, Lijun Hou, Zhipeng Su, Jixi Li

**Affiliations:** 1 Shanghai Pudong Hospital and School of Life Sciences, State Key Laboratory of Genetics and Development of Complex Phenotypes, Shanghai Engineering Research Center of Industrial Microorganisms, Fudan University, Shanghai, China; 2 Department of Neurosurgery, First Affiliated Hospital of Wenzhou Medical University, Wenzhou, China; 3 Department of Neurosurgery, Second Affiliated Hospital of Naval Medical University, Shanghai Institute of Neurosurgery, Shanghai, China; 4 Centre for Immunology and Infection Control, School of Biomedical Sciences, Queensland University of Technology, Brisbane, QLD Australia

**Keywords:** Necroptosis, Cell death in the nervous system

## Abstract

Glioblastoma (GBM), a highly aggressive form of glioma, poses serious harm to patients due to its extremely poor prognosis and severe resistance to chemotherapeutic agents. Although programmed necrosis (necroptosis) has been implicated in GBM progression, its precise function and biological significance in GBM remain incompletely defined. Here, we show that elevated expression of key necroptotic machinery proteins, including RIPK1 and MLKL, is positively associated with disease progression and predicts poor prognosis in glioma patients. Functionally, RIPK1 promotes glioblastoma cell proliferation, migration, and invasion. Genetic ablation of RIPK1 induces cell-cycle arrest and suppresses tumor growth in subcutaneous xenograft models, whereas pharmacological inhibition of RIPK1 with necrostatin-1 fails to restrict GBM cell expansion, suggesting that RIPK1 exerts oncogenic effects independent of its canonical necroptotic role. Notably, dual apoptosis- and necroptosis-inducing agents, ZZW115 and citronellol, synergize with temozolomide (TMZ)—the first-line chemotherapy for GBM—to enhance glioma cell death and increase tumor clearance in an orthotopic mouse glioma model. Collectively, these findings identify RIPK1 as a critical driver of glioma malignancy and underscore the therapeutic potential of activating necroptosis to augment TMZ efficacy, providing a framework for novel prognostic and treatment strategies in glioma.

## Introduction

Glioma is the most prevalent primary malignant brain tumor in adults and has a high disability rate and mortality rate [1]. According to the World Health Organization (WHO) classification, gliomas range from low-grade tumors (grades I–III) with relatively favorable prognosis to the highly aggressive grade IV glioblastoma (GBM) [2–4]. Despite advances in clinical classification based on histopathology and molecular features such as isocitrate dehydrogenase (IDH) status and 1p/19q codeletion [5–7], clinical outcomes for high-grade gliomas remain dismal.

Standard therapy for glioma includes maximal safe surgical resection followed by radiochemotherapy [8, 9]. Yet, GBM patients have a median overall survival of roughly 8 months and a five-year survival rate of approximately 6.8%, with disease recurrence in most patients within 6–9 months [10, 11]. The alkylating agent temozolomide (TMZ) is a cornerstone of glioma treatment, but more than half of patients treated with TMZ eventually develop resistance [3, 4, 6, 10]. Identifying robust prognostic markers and therapeutic strategies to overcome TMZ resistance is thus an urgent clinical priority.

Pathologically, GBM is defined by microvascular hyperplasia, necrosis, and dysregulated apoptosis [10]. The accumulation of necrotic foci within tumors is linked to elevated tumor necrosis factor (TNF) secretion and activation of necroptosis [12], a form of regulated necrotic cell death implicated in the pathogenesis of neurodegenerative disease, infection, and cancer [13]. Canonically, TNF-α stimulation primarily initiates either a pro-survival inflammatory response or triggers the apoptotic pathway [14]. Apoptosis is a highly regulated, caspase-dependent process characterized by cell shrinkage, nuclear fragmentation, and the formation of apoptotic bodies that are phagocytosed without eliciting inflammation. However, various pathological conditions, including viral infection or pan-caspase inhibitors, can suppress the activity of caspase-8 [15]. When this critical apoptotic checkpoint is compromised, cells default to a robust backup death program, necroptosis. In the canonical necroptosis pathway, receptor-interacting protein kinases RIPK1 and RIPK3 form the amyloidal necrosome complex, leading to RIPK3-dependent phosphorylation of MLKL. Phosphorylated MLKL oligomerizes and inserts into the plasma membrane, leading to cellular rupture and the release of damage-associated molecular patterns [16–23]. In cancer therapy, necroptosis can also serve as a mechanism for eliminating therapy-resistant cells [24, 25]. Inhibition of the necroptotic machinery by necrostatin-1 (Nec-1) has been proposed as a therapeutic approach for neurological disease [26]. However, the role of necroptotic machinery in human gliomas is still unknown.

Here, we integrate analyses of RIPK1, RIPK3, and MLKL across clinical glioma samples and experimental models to define the contribution of the necroptotic pathway to glioma pathobiology. Our data identify RIPK1 and MLKL as independent prognostic markers for glioma and reveal distinct roles for RIPK1 beyond its established function in necroptosis. Genetic ablation of RIPK1 or MLKL impairs GBM growth and invasion and promotes cell-cycle arrest. Moreover, combining TMZ with the necroptosis inducers ZZW115 or citronellol significantly improves therapeutic efficacy in orthotopic mouse models, highlighting the potential of targeting necroptosis to overcome therapeutic resistance and improve patient outcomes.

## Materials and methods

### Cell lines and cell culture

The human GBM-derived U87 cell line (RRID: CVCL_0022) was purchased from Beyotime, China (C6981). The U251 cell line (RRID: CVCL_0021) was purchased from Cyagen, China (HCGUI-30001). These human cell lines have been authenticated using STR profiling within the last three years. All cell lines were cultured in Dulbecco’s modified Eagle’s medium (DMEM, BasalMedia, China) supplemented with 10% fetal bovine serum (Lonsera, Uruguay) and 1% penicillin/streptomycin (Gibco, USA), and routinely tested for mycoplasma. All cells were cultured under 37 °C with 5% CO_2_ in a humidified incubator (Thermo Fisher, USA).

For RIPK1 or MLKL knockout, sgRNAs were designed, and the sequences were as follows:

RIPK1-sgRNA: 5′-GGTGATGGAGTACATGGAGA-3′;

MLKL-sgRNA: 5′-GAAGCTGAGTGATGTCTGGA-3′.

The siRNA duplexes transfection was performed using Lipofectamine RNAiMAX (Invitrogen, USA) according to the manufacturer’s protocol. siRNAs were purchased from GenePharma (China), and the sequences were as follows:

si-RIPK1-1: 5′-CCCAGGGACUCAUGAUCAUTT-3′;

si-RIPK1-2: 5′-AUGAUCAUGAGUCCCUGGGTT-3′;

si-NC: 5′-UUCUCCGAACGUGUCACGUTT-3′.

### Clinical glioma samples

This study was reviewed and approved by the Ethics Committee of Wenzhou Medical University, China. Written informed consents were obtained from all patients or their guardians. The authors affirm that human research participants provided informed consent for the publication of this paper. Clinical specimens (16 WHO grade II gliomas, 19 WHO grade III gliomas, and 46 WHO grade IV GBM) were obtained from glioma patients who underwent surgery at the Department of Neurosurgery, First Affiliated Hospital of Wenzhou Medical University, between July 2015 and March 2020. All involved patients were 18 to 77 years old, had detailed clinical history and follow-up information, and had no prior radiotherapy to the brain and no intracranial abscess within 6 months before surgery.

### Data processing and expression analysis

The mRNA expression data of gliomas were downloaded from the TCGA database (http://cancergenome.nih.gov/abouttcga). The respective normal tissue samples were downloaded from the (GTEx, https://gtexportal.org/home/datasets) database. For RNA-seq data, expression levels were TPM-normalized. Expression data for RIPK1, RIPK3, and MLKL were Log2 transformed, and the Wilcoxon rank-sum test was conducted on these tumor types; *p* < 0.05 was considered to indicate differential expression between tumor and normal tissues. Data analysis was conducted using R software (Version 3.6.3), and the R package “ggplot2” was used to draw box plots.

### Survival analysis

The Kaplan-Meier survival analysis for overall survival (OS) of RIPK1/RIPK3/MLKL in different grades of gliomas using R packages “survminer” and “survival” was conducted to compare the survival difference. The relationship between RIPK1/RIPK3/MLKL expression and patients’ DSS and PFI in gliomas was visualized with forest plots. The hazard ratio (HR) and 95% confidence intervals were calculated via univariate survival analysis.

### Analysis of relationships between protein expression and clinical phenotype

Six clinical phenotypes, *IDH* status, *1p/19q* codeletion status, histological type, WHO grade, and primary therapy outcome were selected, and their relationships with RIPK1/RIPK3/MLKL expression in gliomas were explored. In each grade, the association between clinical phenotypes and RIPK1/RIPK3/MLKL expression was analyzed. Clinical phenotype correlation analyses were conducted using R software (Version 3.6.3) with the Kruskal-Wallis test, and the R package “ggplot2” was used to draw box plots.

### Western blotting

Total protein was extracted with a RIPA buffer (50 mM Tris-HCl, pH 7.4, 150 mM NaCl, 1 mM EDTA, 1% Triton X-100, 1% sodium deoxycholate and 0.1% SDS) supplemented with protein inhibitors (Roche, Switzerland). Proteins were separated by electrophoresis through 8–12% polyacrylamide gels. Following the electrophoretic transfer of proteins onto nitrocellulose membranes, non-specific binding was blocked with 5% Bovine Serum Albumin (BSA, Beyotime) for one h at room temperature and incubated in primary antibodies against RIPK1 (Santa Cruz, sc133102), RIPK3 (Santa Cruz, sc374639), MLKL (Abcam, ab184718), p-MLKL (Abcam, ab187091), E-Cadherin (Beyotime, AF0138), N-Cadherin (Beyotime, AF5237), CDK2 (Beyotime, AF1063), Cyclin A2 (Beyotime, AF2524), Cyclin B1 (Beyotime, AF1606), Cyclin E1 (Beyotime, AF2491), cleaved-Caspase-9 (CST, #9505), β-Actin (Proteintech, 66009). Membranes were then washed and incubated with the appropriate horseradish peroxidase-conjugated secondary antibodies (CST, anti-rabbit #7074, anti-mouse #7076). Protein bands were detected using the chemiluminescence system (Tanon, China) and quantified by ImageJ (Fiji) software. Western blotting was performed in three independent experiments.

### Immunohistochemistry and immunofluorescence analysis

For the immunofluorescence analysis, 40 μm free-floating serial coronal frozen sections of human glioma tissues were subjected to an incubation step at 37 °C for 30 min, followed by blocking with a 5% goat serum (Gibco) at 37 °C for an additional 30 min. The sections were then incubated with primary antibodies overnight at 4 °C. Following three washes with phosphate-buffered saline (PBS), species-specific secondary antibodies were applied to the sections, and the resulting fluorescence was visualized using a fluorescence microscopy system (Leica TCS SP8, Germany).

For the immunohistochemistry analysis, the sections were deparaffinized in xylene and rehydrated through a decreasing gradient of ethanol. Endogenous peroxidase activity was blocked by exposing the sections to 3% H_2_O_2_ for 10 min. Heat-mediated antigen retrieval was carried out by subjecting the slides to a sodium citrate buffer (pH 6.0) and heating them twice in a microwave. Next, the sections were blocked with a 5% goat serum (Boster, China) at 37 °C for 45 min. The primary antibodies were then applied to the sections and incubated overnight at 4 °C. After three washes with PBS, corresponding biotin-labeled secondary antibodies were applied to the sections and incubated for 40 min. The sections were then washed in PBS and incubated with a streptavidin-biotinylated complex for 25 min. Immunostaining was achieved using 3′,3′-diaminobenzidine tetrahydrochloride (DAB/H_2_O_2_), and all tissue sections were counterstained with hematoxylin.

### Cell proliferation and colony assays

To determine growth rates, U251 cells were seeded into 96-well culture plate with 4000 cells/well. The number of cells was measured by CCK-8 (APExBIO, K1018). Proliferation curves were illustrated y optical density measurements at 450 nm in a SpectraMax M5 plate reader. For colony assay, U251 cells were seeded on 6-well plates (2000 cells per well) and cultured for the indicated time. At the end of the growth period, cells were fixed with ethanol and stained with a crystal violet solution (Beyotime, C0121). The cell colonies were photographed, and the number and intensity of colonies were counted for statistical analysis.

### Migration and invasion assays

U251 cells were plated on uncoated (for migration assays) or Matrigel-coated (invasion assays) Transwells (Corning, 356230) in a serum-free medium. The serum-supplemented medium was used as a chemoattractant. After incubation at 37 °C for 24–48 h, cells on the upper surface of the filters were removed by cotton swab, and cells that had invaded and migrated to the lower surface of the Transwells were fixed with 4% paraformaldehyde (Beyotime, P0099) and stained using crystal violet solution (Beyotime, C0121). Eight random fields were photographed for each condition, and cells were counted using ImageJ software.

### Bulk RNA sequencing (RNA-seq) and analyses

Total cellular RNAs were isolated using TRIzol reagent (Invitrogen) as per the manufacturer’s instructions. Poly(A) mRNA isolation was then carried out using Oligo(dT) beads. Each sample was amplified through PCR using P5 and P7 primers, and the PCR products were validated. Libraries were prepared according to the manufacturer’s protocol for Illumina NovaSeq 6000 platforms, and sequencing was performed on the Illumina HiSeq instrument. Genes with a log2 fold change (Log_2_FC) > 1 or<–1 and a *p* < 0.05 were considered significantly differentially expressed. Volcano plots, heatmaps, and pathway enrichment analyses were generated using the R packages ggplot2 and heatmap to visualize the differentially expressed genes (DEGs).

### Mice

Female BALB/c nude mice (7 weeks old) were purchased from Cyagen Biosciences Inc. All mice were housed under specific pathogen-free conditions and cared for according to the institutional guidelines on animal care, with the approval of the Scientific Investigation Board of the School of Life Sciences, Fudan University. For subcutaneous injection, 5 × 10^6^ U251 wild-type, RIPK1-KO, or MLKL-KO cells were suspended in 100 μL of serum-free DMEM and then injected subcutaneously into the flanks of mice. No additional randomization was performed prior to this group allocation, and investigators were blinded when assessing the outcome. The tumor size was measured with a vernier caliper and calculated according to the formula: Volume = 1/2 (length × width^2^). When the tumors reached 1800 mm^3^, the mice were sacrificed, and the tumors were collected and photographed.

### Orthotopic GBM model and bioluminescence imaging

7-week-old female nude mice were anesthetized via intraperitoneal (i.p.) injection of 250 mg/kg tribromoethanol (Avertin). A 100% Avertin stock solution was prepared using tert-amyl alcohol, and a fresh 2.5% working solution was made in D‑PBS (without Ca^2+^ or Mg^2+^) immediately before use. Anesthetized mice were secured in a stereotaxic frame in the prone position. The scalp was disinfected with 75% ethanol and then incised sagittally to expose the skull. A 0.5 mm drill bit was used to create a burr hole 2 mm posterior to the bregma and 1.5 mm right of the midline. Using a 10 μL Hamilton syringe mounted on a microsyringe pump, 5 µL of U251‑Luc cell suspension (1 × 10⁶ cells) was injected intracranially at a depth of 3 mm below the dura at 2 µL/min. After a 5‑min pause, the needle was slowly withdrawn to minimize reflux. Post-surgery, animals were warmed on a 37 °C heating pad until recovery and then returned to their cages; no antibiotic prophylaxis was administered. 21, 23, and 25 days after tumor cell implantation, 20 mg/kg TMZ, 3 mg/kg ZZW115, or 10 mg/kg citronellol were i.p. injected into mice.

For in vivo bioluminescence imaging, mice were anesthetized with 250 mg/kg tribromoethanol and received 150 mg/kg D‑luciferin (Beyotime, ST196) i.p. injection. After 10 min, mice were scanned with the NightOWL II LB983 imaging system. Regions of interest analysis and photon flux quantification were performed using the IndiGO software.

### Statistical data

All the data on gene expression was normalized by log2 transformation. A comparison of normal tissues and cancer tissues was performed using the Wilcoxon rank-sum test. The Kruskal-Wallis test was adopted to analyze the associations between clinical phenotypes and expression levels of RIPK1, RIPK3, and MLKL in gliomas. The correlation analysis between the two variables was performed using Spearman’s or Pearson’s test. In survival analysis, the HRs and *p*-value were calculated by univariate Cox regression analysis or the Log-rank test. Kaplan-Meier curves were used to compare the survival of patients stratified according to different levels of RIPK1, RIPK3, and MLKL expression. *p* < 0.05 was set as the significance threshold for all statistical analyses.

Each experiment in a cell or mouse was performed at least three times. Data were analyzed using GraphPad Prism 8.0 and were presented as the mean ± SD. Statistical analysis was performed using Student’s *t* test, one-way ANOVA, or two-way ANOVA. A value of *p* < 0.05 was considered statistically significant.

## Results

### RIPK1, RIPK3, and MLKL are elevated across glioma grades and molecular subtypes

**Fig. 1:**
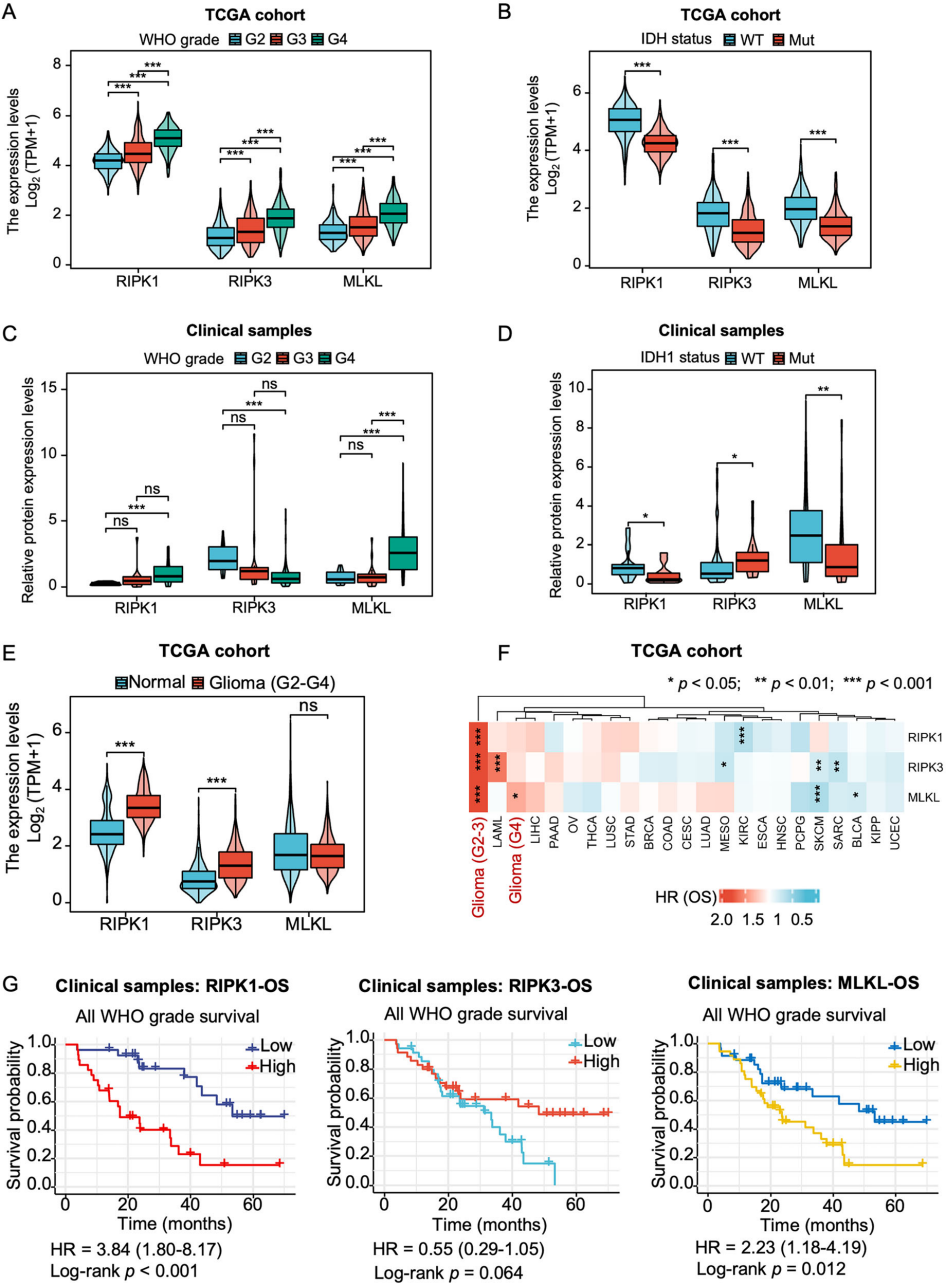
RIPK1 and MLKL expression are associated with poor clinical outcomes in gliomas. mRNA expression levels of RIPK1, RIPK3, and MLKL in the TCGA glioma cohort with WHO grade II–IV (G2–G4, **A**) or IDH mutation status (**B**). **C**, **D** Protein levels of RIPK1, RIPK3, and MLKL in 81 clinical glioma samples grouped by 1p/19q codeletion status. **E** mRNA expression of RIPK1, RIPK3, and MLKL in gliomas (G2–G4) versus normal brain tissue. **F** Heatmap of hazard ratios (HRs) for overall survival (OS) comparing high versus low expression of RIPK1, RIPK3, and MLKL across 23 cancer types. **G** Kaplan-Meier OS curves (High vs. Low) for patients stratified by different protein levels of RIPK1, RIPK3, or MLKL in gliomas. The 81 patients with G2–G4 gliomas were reviewed retrospectively, and protein expression levels were collected from Fig. S2. Data are presented as mean ± SD. Comparisons between two groups were performed using the Wilcoxon rank-sum test. *p < 0.05, **p < 0.01, ***p < 0.001, ns no significance.

To investigate the role of the necroptotic machinery in glioma, we analyzed the mRNA expression profiles of RIPK1, RIPK3, and MLKL across normal brain and glioma samples. Analysis of The Cancer Genome Atlas (TCGA) and Genotype-Tissue Expression (GTEx) databases revealed a significant upregulation of RIPK1 and RIPK3 mRNAs in grade II–IV gliomas (G2–G4) compared with normal brain tissue (Fig. 1A). Similar results were observed in the Chinese Glioma Genome Atlas (CGGA) dataset, where mRNA levels of RIPK1, RIPK3, and MLKL were elevated across glioma grades (Fig. S1A). In both TCGA and CGGA cohorts, tumors with wild-type IDH exhibited higher expression of RIPK1, RIPK3, and MLKL relative to IDH-mutant gliomas (Figs. 1B and S1B), consistent with the more aggressive clinical behavior associated with IDH-wild-type status [27].

We next evaluated protein expression profiles using data from the Clinical Proteomic Tumor Analysis Consortium, which confirmed elevated RIPK1, RIPK3, and MLKL protein abundance in GBM relative to normal tissue (Fig. S1C). To validate these findings, we performed Western blot analyses across a clinical cohort of 81 glioma samples (16 grade II, 19 grade III, and 46 grade IV) (Fig. S2), which revealed significant upregulation of RIPK1 and MLKL and downregulation of RIPK3 in grade IV tumors relative to lower-grade gliomas (Fig. 1C). Notably, RIPK1 appeared predominantly in its cleaved form across glioma samples, suggesting loss of its death-promoting activity and a shift toward pro-survival signaling (Fig. S2). In line with mRNA findings, RIPK1 and MLKL protein levels were higher in IDH-wild-type gliomas compared with IDH-mutant tumors (Fig. 1D). Whereas RIPK1 and RIPK3 transcript levels were elevated across grades II–IV relative to normal tissue (Fig. 1E), all three genes were significantly upregulated in grade IV tumors (Fig. S3A). Consistently, tumors with 1p/19q codeletion—a molecular signature associated with better prognosis—displayed lower mRNA levels of RIPK1, RIPK3, and MLKL (Fig. S3B–F). Interestingly, the discordance between RIPK3 mRNA and protein expression suggests post-transcriptional or post-translational regulation. Collectively, these data highlight the association between necroptosis machinery and glioma aggressiveness, suggesting a pivotal role for RIPK1, RIPK3, and MLKL in disease progression and clinical outcome.

### High expression of RIPK1, RIPK3, and MLKL predicts poor prognosis in glioma

To assess the prognostic significance of the necroptotic genes RIPK1, RIPK3, and MLKL in gliomas, we performed survival analyses across multiple datasets. A Cox proportional hazards model applied to 23 TCGA cancer cohorts identified RIPK1, RIPK3, and MLKL as significant risk factors for OS in WHO grade II and III gliomas (Fig. 1F). Kaplan–Meier analyses of 703 glioma patients in TCGA revealed that while expression of RIPK1, RIPK3, and MLKL did not correlate with OS in grade II tumors, high expression of each was associated with significantly reduced OS in grade III gliomas (HR = 2.03, *p* = 0.002; HR = 2.09, *p* = 0.002; HR = 2.10, *p* = 0.001, respectively) (Fig. S4A–C). Consistently, high expression of these genes was associated with worse disease-specific survival and progression-free interval in all WHO grades (Fig. S4D–F). Similar findings were observed in the CGGA dataset, where RIPK1, RIPK3, and MLKL served as independent prognostic indicators across all WHO grades and within grade III gliomas (Fig. S5). In the TCGA cohort, elevated MLKL expression was linked with poor OS in GBM (Fig. S4C), while CGGA data identified high RIPK3 expression as a risk factor in the same disease context (Fig. S5B). Immunoblot analyses of clinical samples further confirmed that elevated protein levels of RIPK1 and MLKL were associated with shorter patient survival (Fig. 1G). Together, these findings demonstrate that the necroptotic genes RIPK1, RIPK3, and MLKL are robust predictors of adverse clinical outcomes across glioma subtypes.

### RIPK1 and MLKL drive glioma proliferation and tumorigenesis

**Fig. 2:**
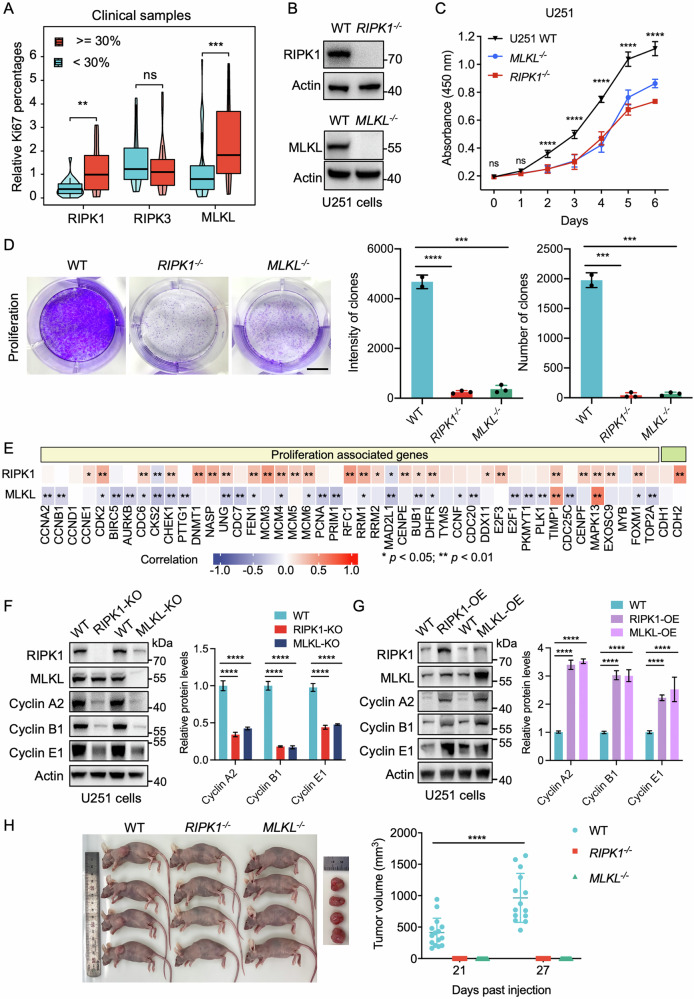
Depletion of RIPK1 or MLKL inhibits glioma proliferation in vitro and in vivo. **A** Ki-67 staining in glioma tissues stratified by RIPK1 or MLKL protein expression levels, which are quantified from Fig. S2. **B** Immunoblot analysis of RIPK1 and MLKL in wild-type (WT), RIPK1 knockout (KO), and MLKL-KO U251 cells. **C** CCK-8 assay showing reduced proliferation of RIPK1-KO and MLKL-KO U251 cells compared to WT controls. **D** U251 cells were seeded on 6-well plates, and the colony formation assays were visualized on day 14. Scale bar, 10 mm. **E** Correlation between RIPK1/MLKL mRNA expression and genes associated with proliferation and EMT in TCGA GBM cohort. **F** Immunoblots showing endogenous protein expression from wild-type (WT) or RIPK1 knockout (KO) U251 cells. **G** Immunoblots showing endogenous protein expression from WT, RIPK1 overexpression (OE) or MLKL-OE U251 cells. **H** Tumor images and quantification of tumor volume in xenograft models implanted with WT, RIPK1-KO, or MLKL-KO U251 cells (*n* = 14). Data are presented as mean ± SD. Statistical significance was determined using the Wilcoxon rank-sum test (**A**), two-way ANOVA (**C**), or Student’s *t* test (**F**–**H**). *p < 0.05, **p < 0.01, ***p < 0.001, ns no significance.

To investigate the role of RIPK1 and MLKL in glioma progression, we first examined their expression in relation to proliferative activity. In a cohort of 81 clinical glioma samples, high Ki67 labeling indices (30–80%) were associated with significantly elevated RIPK1 and MLKL expression compared to low-Ki67 tumors (<30%) (Fig. 2A). Since Ki67 is a well-validated marker of cellular proliferation [28, 29], this finding suggests that RIPK1 and MLKL may contribute to the aggressive behavior of glioma. Consistently, siRNA-mediated knockdown of RIPK1 or MLKL suppressed the proliferation of U87 cells (Fig. S6A). In U251 glioblastoma cells, CRISPR-Cas9-mediated ablation of RIPK1 or MLKL impaired cellular growth, as demonstrated by CCK-8 and colony-formation assays (Fig. 2B–D). To assess the association between RIPK1 or MLKL expression and cellular proliferation and invasion, we analyzed TCGA transcriptomic data for a panel of known markers [30]. RIPK1 was positively correlated with the expression of cyclin E1 (CCNE1), CDK2, TIMP1, N-cadherin (CHD2), and other genes implicated in glioma proliferation and invasion [30–32], whereas MLKL exhibited a weaker or negative correlation with most genes in this signature (Fig. 2E). These observations support a more dominant role for RIPK1 in driving glioma growth.

Western blot analyses confirmed that RIPK1 or MLKL loss suppressed the expression of cyclin A2, cyclin B1, and cyclin E1 in U251 cells, whereas overexpression of either protein restored the levels of these cyclins (Fig. 2F, G). Notably, RIPK1 expression in *MLKL*^−/−^ U251 cells is significantly lower compared to WT cells. This observation raises the possibility that MLKL loss alters upstream necroptotic or protein-stability networks that converge on RIPK1. Moreover, MLKL ablation may, at least in part, influence glioma cell-cycle progression through effects on RIPK1. However, further studies are required to validate this hypothesis. In vivo, implantation of *RIPK1*^−^^/−^ or *MLKL*^−^^/−^ U251 cells into nude mice failed to form subcutaneous tumors, in sharp contrast to robust tumor growth observed for the corresponding wild-type controls (Fig. 2H). Collectively, these results demonstrate that both RIPK1 and MLKL critically support glioma cell proliferation and tumorigenesis, with RIPK1 exerting a more potent role.

### RIPK1 promotes glioma cell cycle progression independent of its kinase activity

**Fig. 3:**
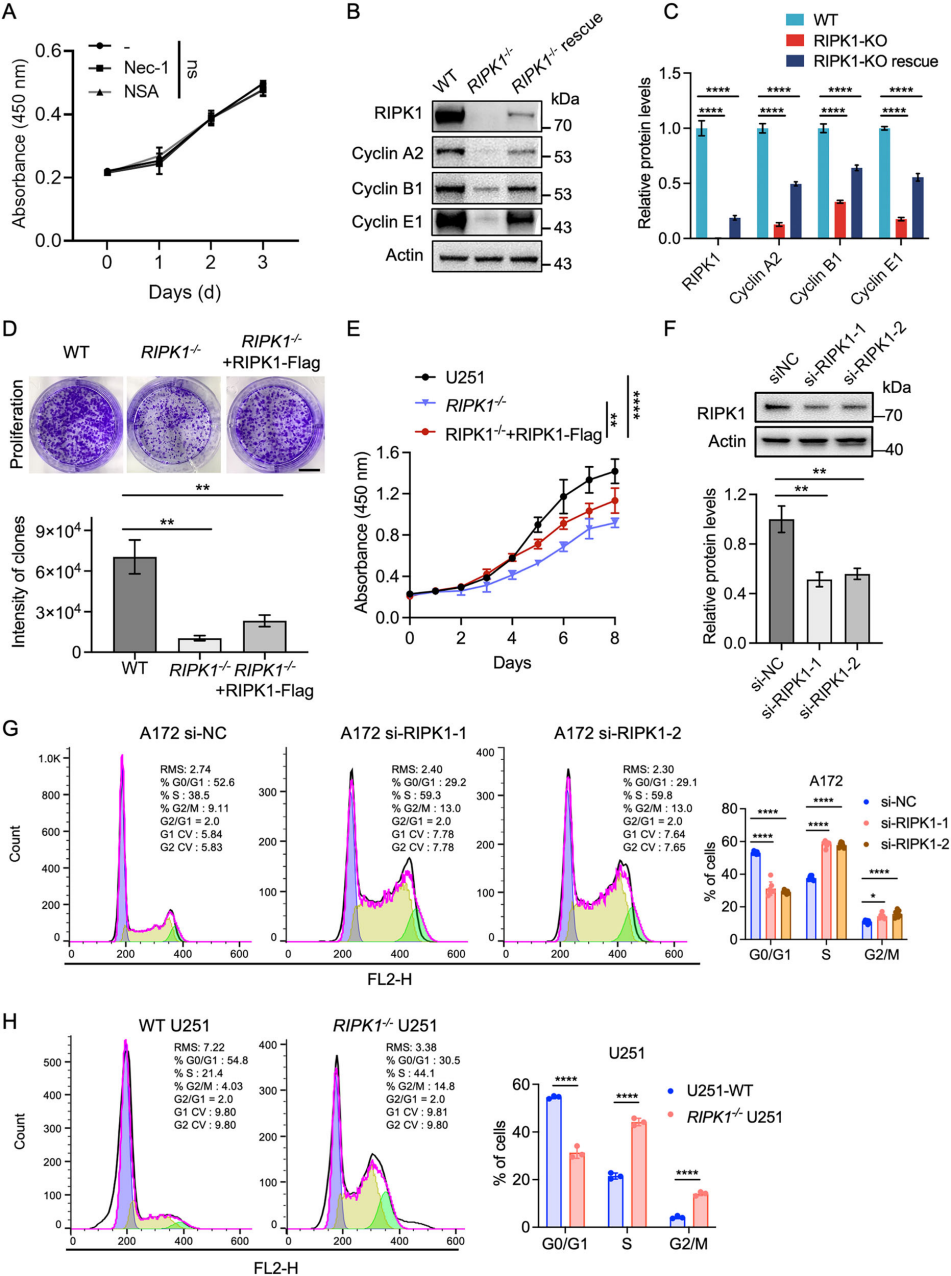
RIPK1 knockout arrests cell cycle progression. **A** The cell proliferation rate of U251 cells treated with RIPK1 inhibitor (Nec-1, 10 μM) or MLKL inhibitor (NSA, 10 μM) for 12 h, as determined by the CCK-8 assay. **B** Immunoblots of the indicated protein expression in WT U251 cells, RIPK1-KO U251 cells, and RIPK1-KO cells rescued with RIPK1-Flag. **C** Quantitative analysis of the relative protein levels in (**B**). **D** U251 cells were seeded on 6-well plates and the colony formation assays were visualized on day 14. Scale bar, 10 mm. **E** Cell proliferation rate of U251 cells with different genotypes, as measured by the CCK8 assay. **F** Immunoblot and densitometric quantification of cell cycle-related proteins in A172 cells 36 h post-transfection with negetive control (NC) or RIPK1-targeting siRNAs. Flow cytometry analysis of cell cycle distribution in A172 (**G**) and U251 (**H**) cells following RIPK1 depletion. Data are presented as mean ± SD. Statistical analysis was performed using the Student’s *t* test. *p < 0.05, **p < 0.01, ***p < 0.001; ns no significance.

RIPK1 depletion significantly reduced glioma cell proliferation, prompting an investigation into the role of its kinase activity. Treatment of U251 glioma cells with the RIPK1 kinase inhibitors Nec-1 or the MLKL oligomerization and membrane translocation inhibitor necrosulfonamide (NSA), respectively, had no significant impact on cellular growth (Fig. 3A), indicating that the pro-proliferative role of RIPK1 is kinase-independent. In RIPK1-knockout U251 cells, the expression of key cyclins was diminished (Fig. 2F), including cyclin A2 (a regulator of S-phase progression and Cdk2 activity), cyclin B1 (a mediator of mitotic entry via Cdk1), and cyclin E1 (a trigger for G1-to-S transition) [33–35]. Importantly, re-expression of RIPK1 in the knockout cells restored the protein levels of cyclin A2/B1/E1 (Fig. 3B, C). Furthermore, this re-expression also rescued the impaired cell proliferation rate and colony-formation ability (Fig. 3D, E). Consistently, flow cytometric analyses of U251 and A172 GBM lines revealed that loss of RIPK1 promoted S- and G2/M-phase arrest (Fig. 3F–H). These data indicate that RIPK1 supports glioma proliferation by maintaining cyclin expression and promoting progression through critical checkpoints of the cell cycle, independent of its canonical necroptotic kinase activity.

### RIPK1 promotes glioma cell migration and invasion via EMT and ECM modulation

**Fig. 4:**
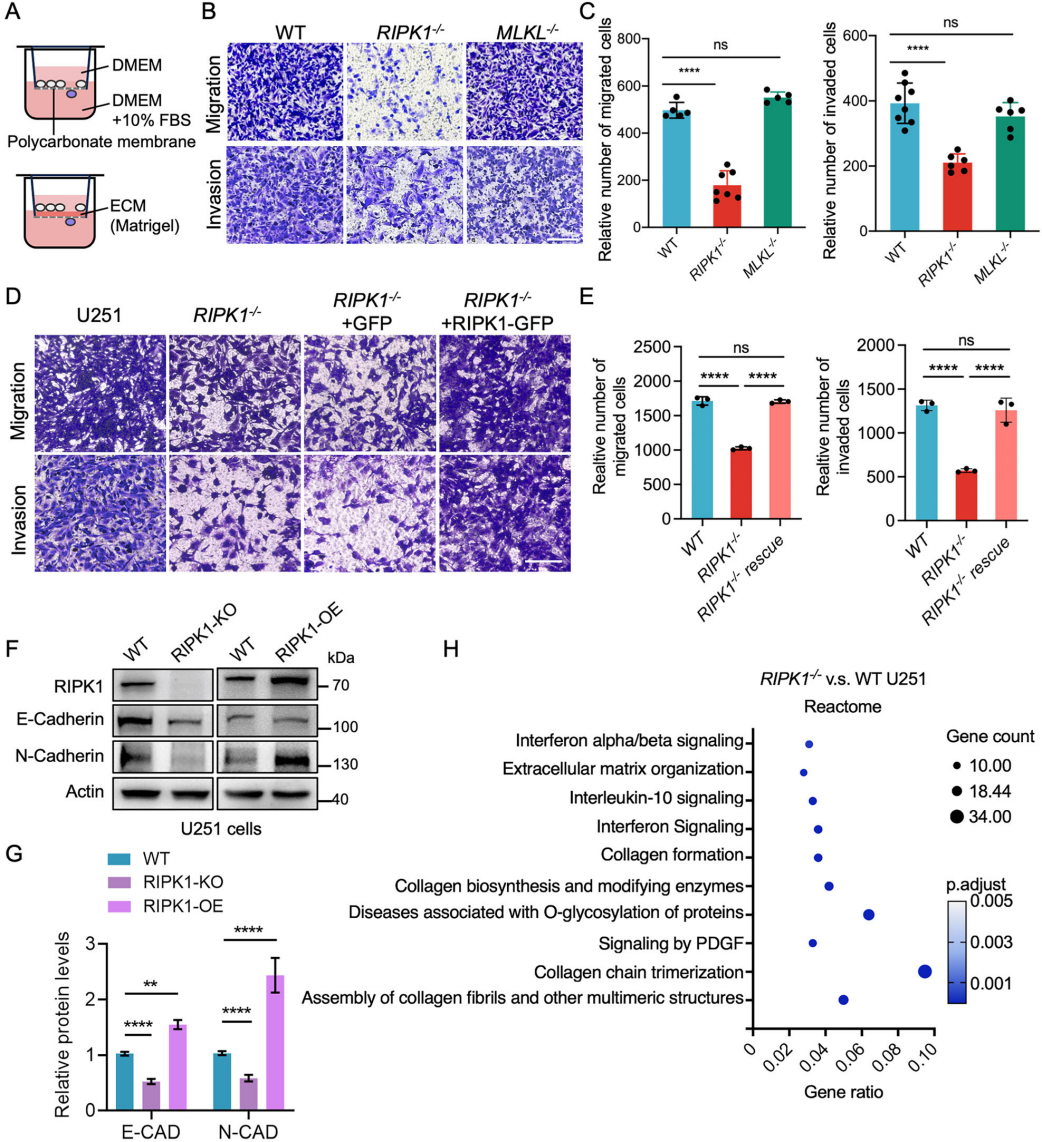
RIPK1 knockout suppresses glioma cell migration and invasion in vitro. **A** Schematic overview of transwell-based migration and invasion assays. ECM extracellular matrix. **B**, **C** Transwell assays showing reduced migration and invasion in *RIPK1*^−^^/^^−^ and *MLKL*^*−/−*^ U251 cells compared to wild-type controls. Scale bar, 100 μm. **D**, **E** Rescue experiments using plasmid transfection in WT and *RIPK1*^*−/−*^ U251 cells followed by transwell assays. Scale bar, 100 μm. **F** Immunoblot analysis of migration- and EMT-related proteins in WT, RIPK1-knockout (KO), and RIPK1-overexpressing (OE) U251 cells. **G** Quantification of protein expression in (**F**). **H** REACTOME pathway enrichment analysis of differentially expressed genes in *RIPK1*^*−/−*^ U251 cells, highlighting alterations in signaling and migration-associated pathways. All experiments were performed in triplicate. Error bars represented means ± SD. *p < 0.05, **p < 0.01, ***p < 0.001, ****p < 0.0001, ns no significance.

To investigate the role of RIPK1 and MLKL in glioma invasiveness, we performed Transwell migration and Matrigel invasion assays in U251 cells. Deletion of RIPK1 significantly reduced the number of migrating and invading cells, whereas MLKL loss had no discernible effect (Fig. 4A–C). Importantly, re-expression of RIPK1 in RIPK1-null U251 cells restored both migratory and invasive capabilities (Fig. 4D, E). Consistently, RIPK1 knockout reduced N-cadherin expression, a hallmark of epithelial–mesenchymal transition (EMT) in glioma [36, 37], while RIPK1 overexpression increased its expression (Fig. 4F, G), suggesting that RIPK1 promotes an EMT-like phenotype.

To define the molecular pathways underpinning RIPK1-dependent glioma invasiveness, we conducted RNA-seq analyses of wild-type versus *RIPK1*^–/–^ U251 cells. Differential expression analysis identified 175 downregulated and 553 upregulated genes upon RIPK1 loss (FDR-adjusted *p* < 0.05, |log_2_FC| > 1; Fig. S6B). Reactome pathway enrichment revealed that downregulated genes were associated with extracellular matrix organization and collagen formation (Fig. 4H). Cell adhesion-related genes were specifically visualized in Fig. S6C, with the top differentially expressed genes highlighted. Similar findings were observed in a published RNA-seq dataset comparing *Ripk1*^*–/–*^ MEFs and controls [38], where KEGG and GO analyses implicated RIPK1 in collagen-rich ECM and cell adhesion molecule binding (Fig. S6D). Together, these results indicate that RIPK1 promotes glioma invasiveness by facilitating EMT and shaping the collagen-rich extracellular microenvironment.

### Necroptosis induction sensitizes glioma to temozolomide therapy

**Fig. 5:**
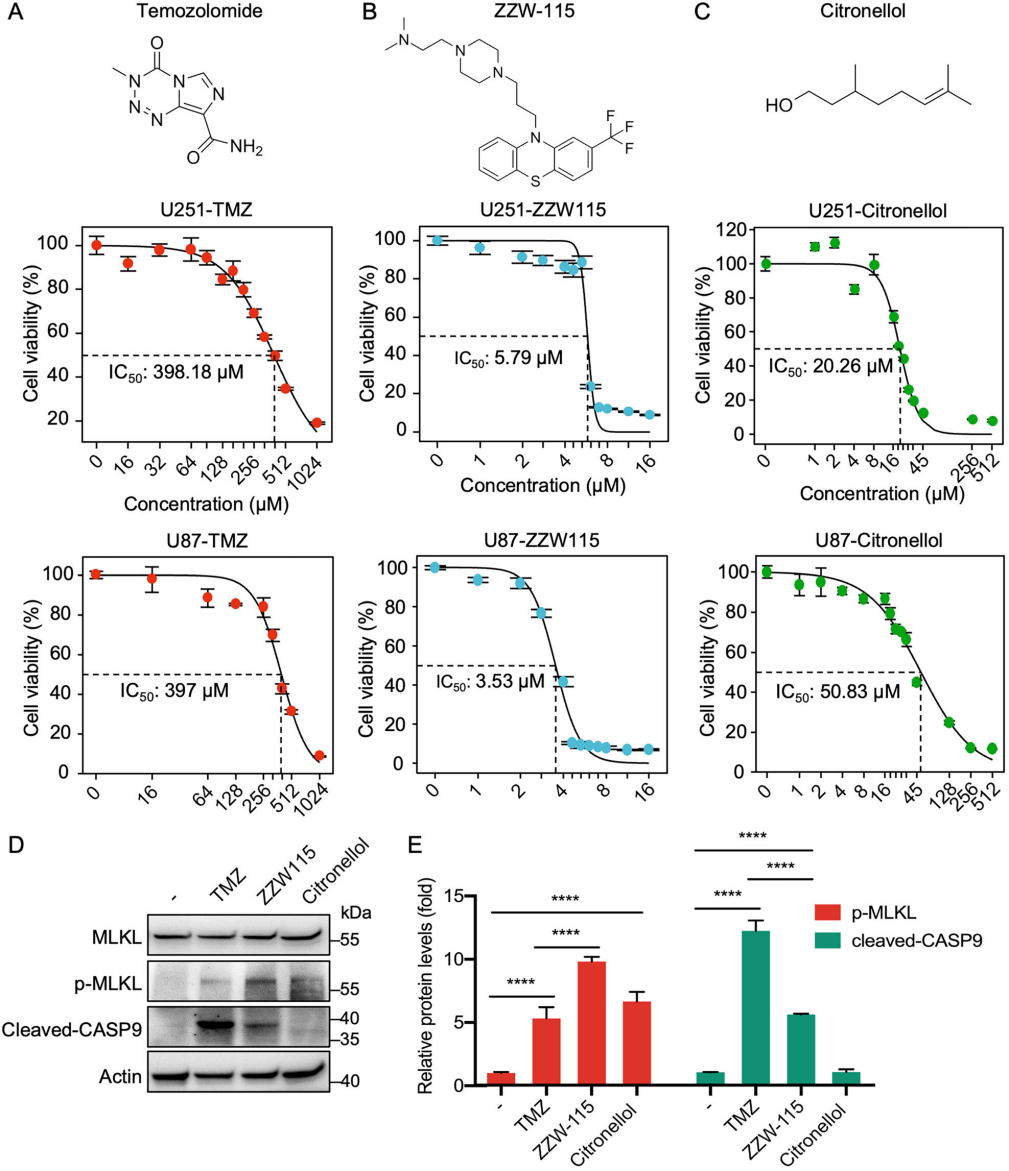
Necroptosis inducers ZZW115 and Citronellol exhibit enhanced cytotoxicity against glioblastoma cells. Chemical structures of standard chemotherapeutic agents and necroptosis inducers (**A**), and dose–response curves for U251 and U87 cells treated with these compounds (**B**, **C**). The half-maximal inhibitory concentration (IC₅₀) values are indicated. **D** Western blot analysis of U251 cells treated with TMZ, ZZW115, or Citronellol for 48 h at IC₅₀ concentrations. **E** Quantification of protein expression from immunoblots shown in (**D**), highlighting necroptotic pathway activation. Error bars represented means ± SD. *p < 0.05, ***p < 0.001, ****p < 0.0001, ns no significance.

TMZ is a first-line treatment for GBM, exploiting its ability to cross the blood–brain barrier and kill MGMT-silenced tumors via apoptotic and autophagic pathways [39]. However, more than 50% of patients eventually develop resistance, often due to MGMT overexpression, enhanced DNA repair, the presence of glioma stem-like cells, and activation of the prosurvival pathway [39]. Necroptosis, an alternative form of regulated cell death, has emerged as a promising approach for bypassing apoptotic resistance [40, 41]. In this context, we examined the effects of two necroptosis-inducing agents, ZZW-115 and citronellol, in glioma models. ZZW-115, an antitumor agent that triggers both apoptosis and necroptosis, and citronellol, a natural compound that promotes RIPK1/RIPK3-dependent necroptosis [42, 43], were tested in U251 and U87 glioma lines (Fig. 5A–C). Notably, ZZW-115 reduced glioma cell viability with an IC_50_ of 5.79 μM (U251 cells) and 3.53 μM (U87 cells), making it roughly 60-fold more potent than TMZ (Fig. 5B). Similarly, citronellol was approximately ten times more effective than TMZ in U251 cells (Fig. 5C).

**Fig. 6:**
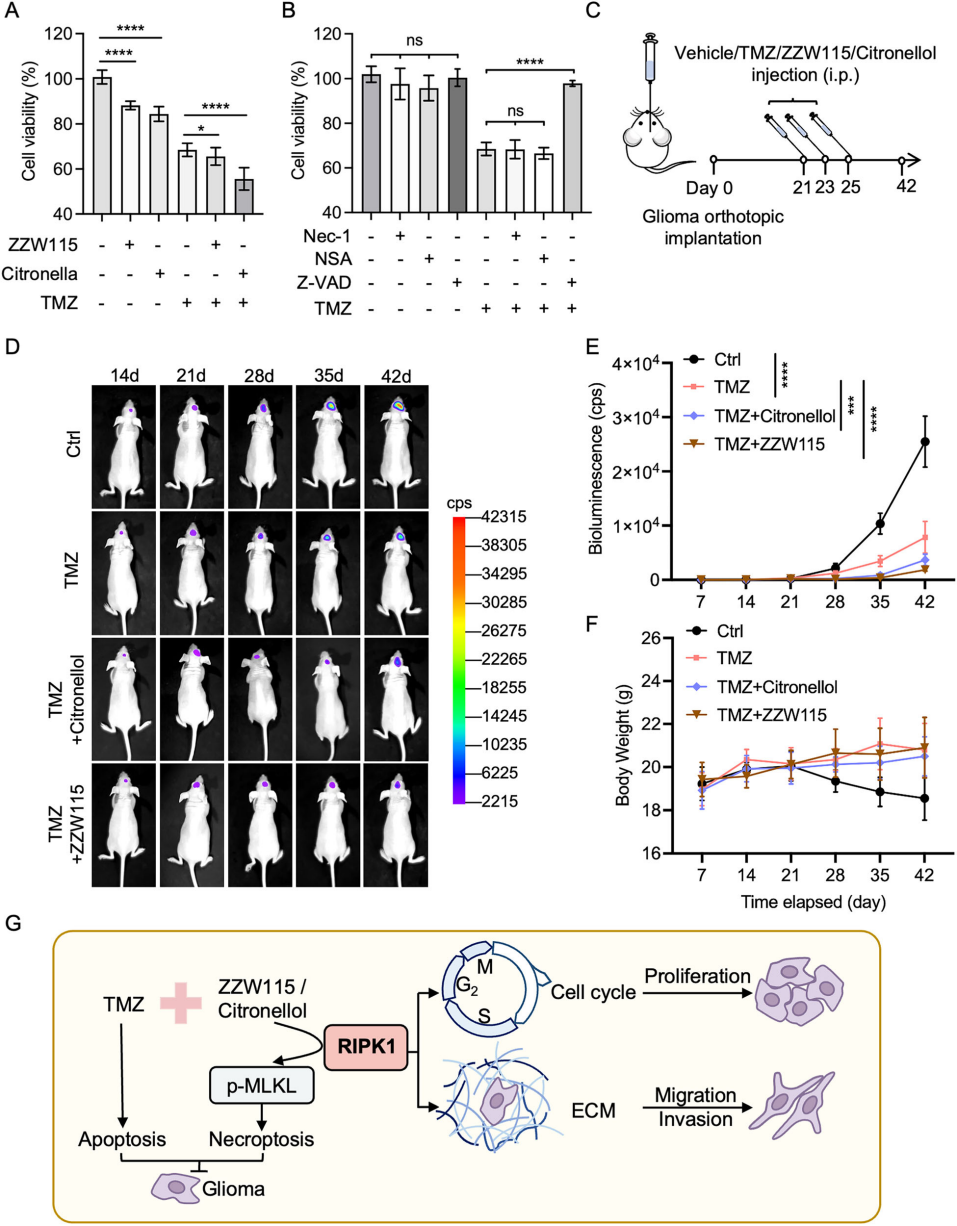
Dual induction of necroptosis and apoptosis suppresses glioma growth in vivo. **A**, **B** U251 cells were treated with 200 μM temozolomide (TMZ) alone or in combination with necroptosis inducers/inhibitors—4 μM ZZW115, 10 μM citronellol, 10 μM Nec-1, 10 μM NSA or 20 μM Z-VAD—for 48 h. Cell viability was determined using the CCK-8 assay. **C** Schematic of the glioma orthotopic implantation model. Luciferase-expressing U251 cells were injected into the brains of nude mice, followed by intraperitoneal drug administration on days 21, 23, and 25. **D** Representative bioluminescence images at the indicated time points show tumor progression in each treatment group (*n* = 5 mice per group). **E** Quantification of tumor burden based on weekly bioluminescence imaging. **F** Body weight monitoring of mice from each treatment group throughout the experiment. **G** Graphical summary illustrating the role of RIPK1 in glioma malignancy. Combination therapy inducing necroptosis and apoptosis enhances TMZ-mediated tumor suppression. ECM, extracellular matrix. The data are the means ± SD of triplicate samples from a representative experiment. * *p* < 0.05, ** *p* < 0.01, *** *p* < 0.001, **** *p* < 0.0001, ns no significance.

Western blot analysis revealed that TMZ treatment predominantly activated caspase-9 and induced apoptosis, while ZZW-115 and citronellol treatments increased the expression level of p-MLKL, a hallmark of necroptosis (Fig. 5D, E). Notably, combining TMZ with ZZW-115 or citronellol significantly enhanced U251 cell death relative to TMZ alone (Fig. 6A). In contrast, pharmacological necroptosis inhibition by Nec-1 (a RIPK1 kinase inhibitor) or NSA (a MLKL oligomerization inhibitor) failed to affect TMZ’s cytotoxic effect (Fig. 6B). Meanwhile, the pan-caspase inhibitor Z-VAD rescued the apoptosis induced by TMZ, confirming that TMZ primarily triggers caspase-dependent apoptosis. To validate these findings in vivo, we established orthotopic U251 xenografts in nude mice (Fig. 6C). Co-treatment with TMZ and either ZZW-115 or citronellol achieved superior tumor clearance relative to TMZ alone, as evidenced by bioluminescence imaging (Fig. 6D, E), with no significant changes in body weight (Fig. 6F). Together, these results highlight the therapeutic potential of necroptosis induction to overcome TMZ resistance and improve treatment outcomes in glioma.

## Discussion

Gliomas are the most common and aggressive primary brain tumors in adults, with GBMs presenting significant therapeutic challenges due to their resistance to apoptosis and highly invasive behavior [44, 45]. Although necroptosis has emerged as a promising therapeutic target for apoptosis-resistant tumors [46, 47], its role in gliomas has remained poorly defined. In this study, we demonstrate that the necroptotic machinery genes RIPK1, RIPK3, and MLKL are highly expressed in gliomas and positively correlate with disease progression and patient prognosis (Figs. 1 and S1). Elevated RIPK1 protein levels correlate with IDH1 wild-type status and increased Ki67 expression, aligning with its association with glioma aggressiveness and correspondingly demonstrating the highest HR for reduced OS (Figs. 1D, G and 2A).

Functionally, RIPK1 ablation suppresses GBM cell growth both in vitro and in vivo, leading to a significant downregulation of key cyclins (cyclin A2, B1, and E1) and arrest of the cell cycle at the S and G₂/M phases (Figs. 2F–3G and 3B, C). Notably, these effects occur independently of RIPK1’s kinase activity (Fig. 3A), highlighting its kinase-independent role in regulating glioma cell proliferation. Importantly, our findings link RIPK1 to glioma invasiveness, as its loss significantly reduces migratory and invasive capabilities and downregulates N-cadherin expression (Fig. 4A–G). RNA-seq and pathway analyses further support this role, implicating RIPK1 in collagen formation and extracellular matrix organization (Figs. 4H and S6D). Together, these results establish RIPK1 as a central node coordinating both the proliferative and invasive phenotypes of glioma.

Our results also highlight the therapeutic potential of leveraging necroptosis in GBM. Previous studies have implicated necroptosis in sensitizing tumors to therapy [46], and our data demonstrate that the necroptosis inducers ZZW-115 and citronellol can synergize with TMZ to potentiate its antitumor effects in glioma models (Fig. 6). Though further optimization of these compounds is required, their ability to overcome resistance to TMZ provides a strong rationale for clinical exploration.

In summary, this study identifies RIPK1 and MLKL as critical drivers of glioma progression and prognostic biomarkers. By acting as kinase-independent regulators of the cell cycle and mediators of invasion, these necroptotic genes enable glioma malignancy and therapy resistance. Targeting the necroptotic pathway may thus open new avenues for treating this devastating disease and form the foundation for future therapeutic strategies (Fig. 6G).

## Supplementary information

Supplementary Materials

Original Western blots

## Data Availability

The raw data of RNA-seq data generated in this study have been deposited in the NCBI Sequence Read Archive under the accession number PRJNA1279353. All data needed to evaluate the conclusions in the paper are present in the paper and/or the Supplementary Materials. Additional data related to this paper may be requested from the lead contact, Jixi Li (lijixi@fudan.edu.cn).
