# Supplementary material for: Necroptotic signaling orchestrates glioblastoma malignancy and potentiates temozolomide response
Source: Cell Death Dis. 2025 Dec 22;16(1):921. doi: 10.1038/s41419-025-08377-3 (PMC12748541; doi:10.1038/s41419-025-08377-3)
Supplement: Supplementary file 1 — Supplementary Materials [file 41419_2025_8377_MOESM1_ESM.docx]

**Supplementary Materials**


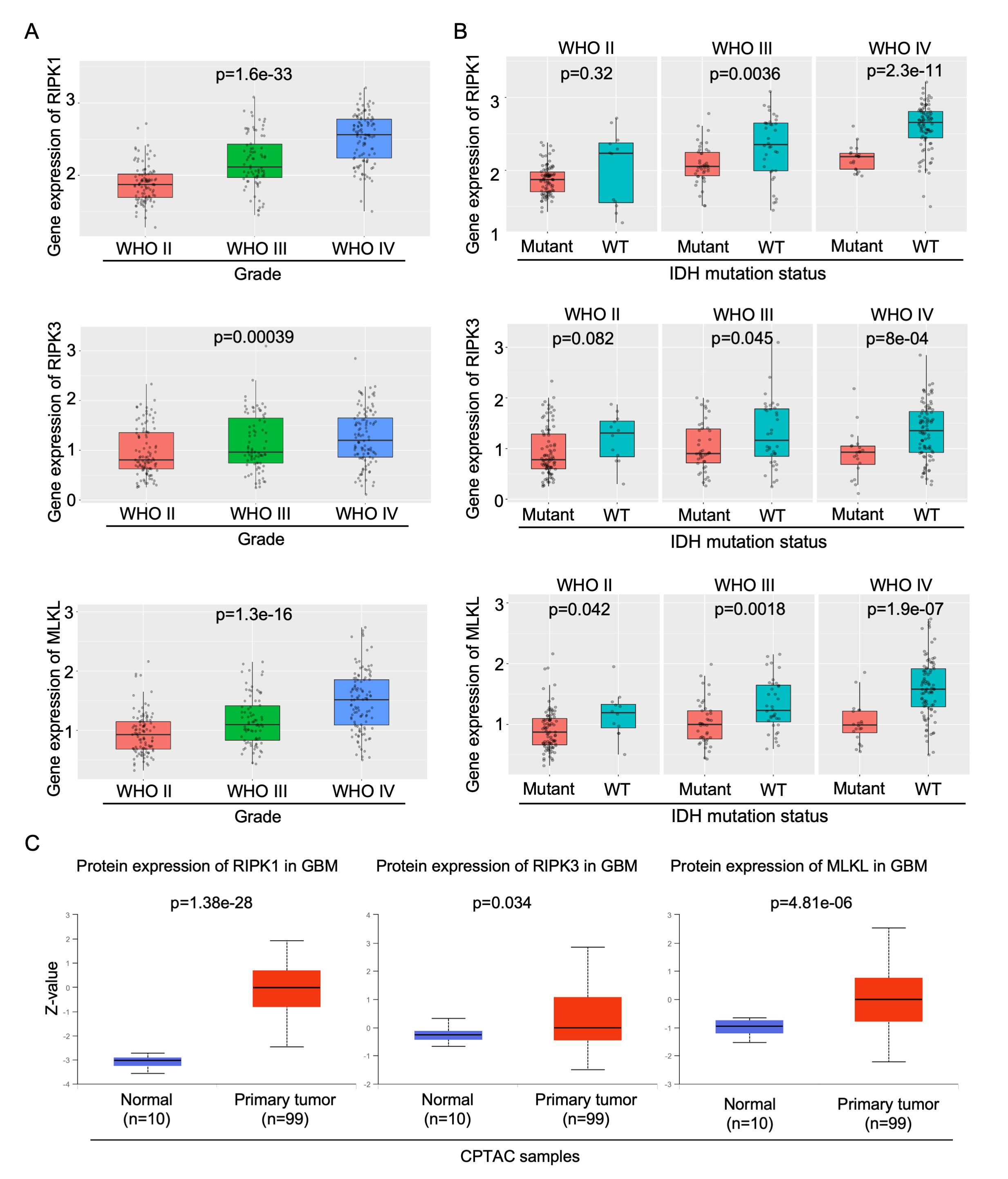


**Figure S1. Elevated RIPK1, RIPK3, and MLKL expression in gliomas with distinct IDH mutation status.**
(**A–C**) RIPK1, RIPK3, and MLKL mRNA expression in gliomas stratified by IDH mutation status in the CGGA cohort. (**D**) RIPK1, RIPK3, and MLKL protein levels in normal brain tissues and primary GBM tissues based on CPTAC datasets.

**Figure S2. Protein expression of RIPK1, RIPK3, and MLKL in clinical glioma tissues.**
(**A-B**) Western blot analysis of RIPK1 (**A**), RIPK3, and MLKL (**B**) in human glioma samples. WHO grade (Ⅱ–Ⅳ), patient gender (F, female; M, male), and IDH1 status (M, mutant; W, wild-type; blank, unavailable) are indicated above each lane; 1p and/or 19q deletion status not shown.


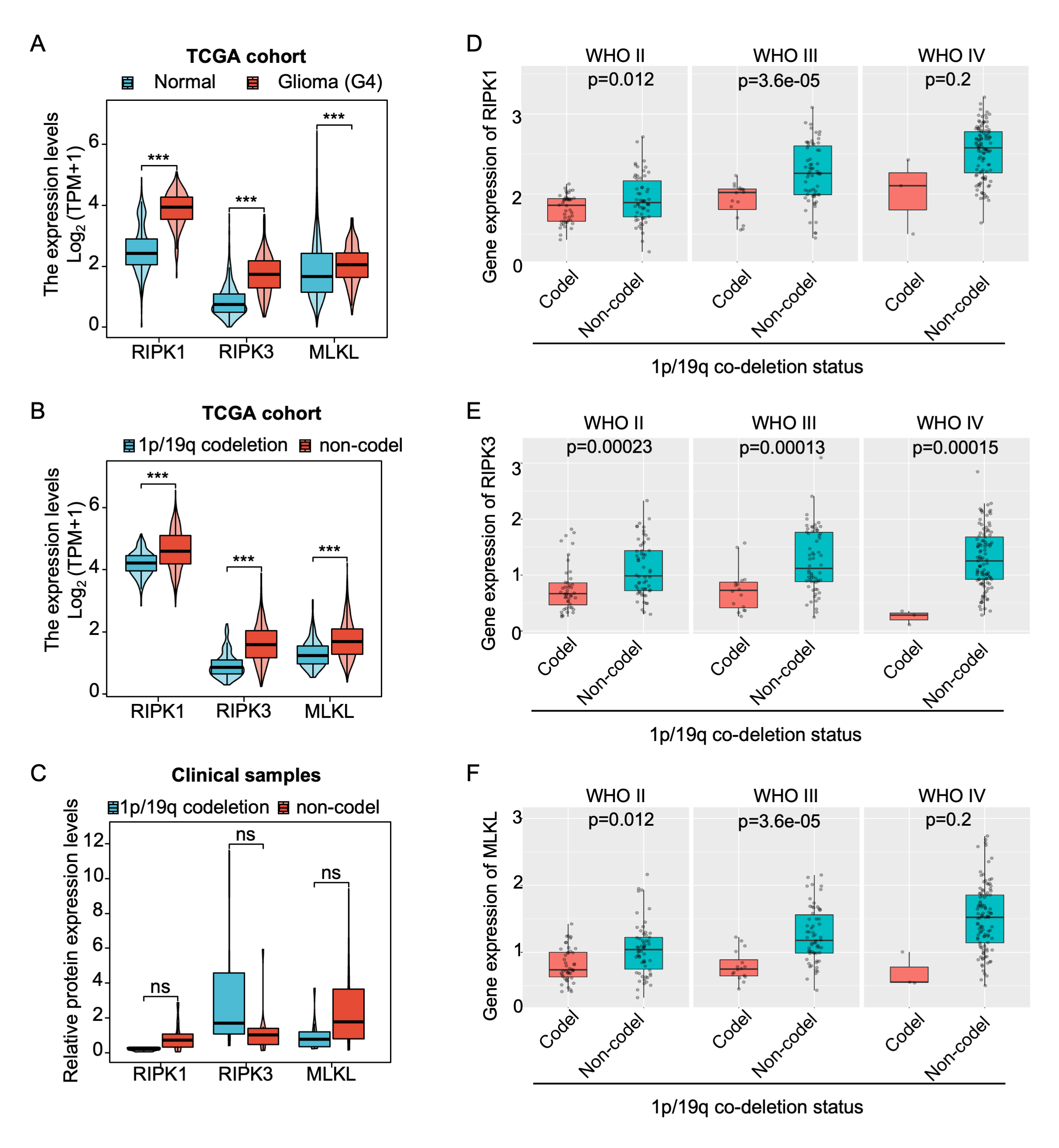


**Figure S3. RIPK1, RIPK3, and MLKL expression in gliomas stratified by 1p/19q codeletion status.**(**A**) mRNA expression of RIPK1, RIPK3, and MLKL in GBM (WHO grade IV) and normal brain tissues. (**B**) mRNA expression of RIPK1, RIPK3, and MLKL in gliomas with different 1p/19q codeletion status. (**C**) Protein expression levels of RIPK1, RIPK3, and MLKL in 81 clinical glioma tissues grouped by 1p/19q codeletion status. (**D–F**) mRNA levels of RIPK1, RIPK3, and MLKL in gliomas with different 1p/19q codeletion status based on the CGGA cohort.


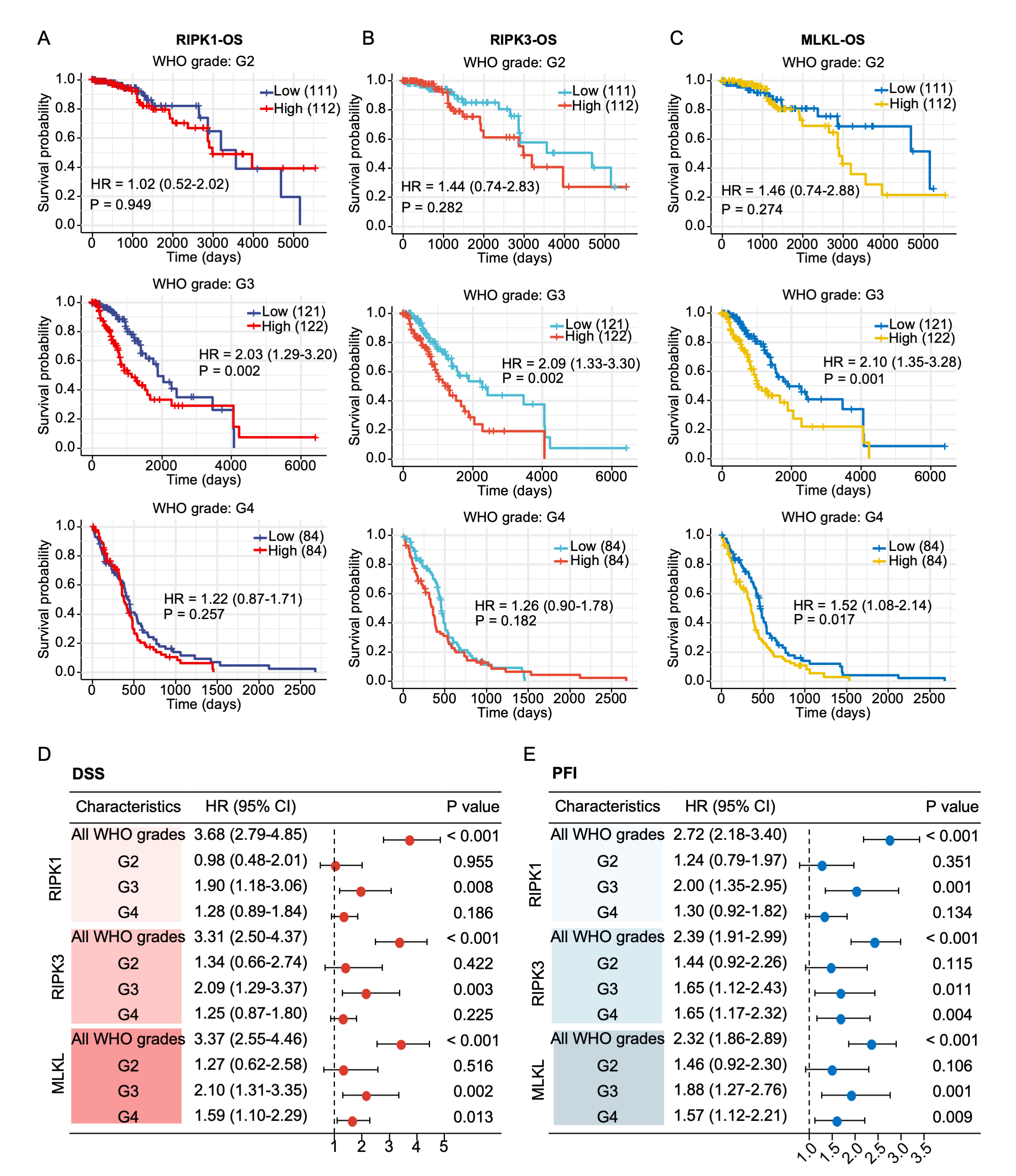


**Figure S4. Prognostic value of RIPK1, RIPK3, and MLKL expression in glioma patients.**(**A–C**) Kaplan–Meier overall survival (OS) curves for glioma patients stratified by high versus low expression of RIPK1 (**A**), RIPK3 (**B**), or MLKL (**C**) in the TCGA cohort. (**D-E**) Forest plots showing the associations of RIPK1, RIPK3, and MLKL expression with disease-specific survival (DSS) (**D**) and progression-free interval (PFI) (**E**) in gliomas of different WHO grades. Hazard ratios (HRs), 95% confidence intervals (CIs), and p values are indicated. **p* < 0.05, ***p* < 0.01, ****p* < 0.001.

**Figure S5. RIPK1, RIPK3, and MLKL gene expression were correlated with OS in the CGGA database.**

(**A-C**). The high- and low-expression values of RIPK1 (**A**), RIPK3 (**B**), or MLKL (**C**) were compared by Kaplan-Meier survival curves for the CGGA glioma cohort with different WHO grades. The horizontal axis indicated the overall survival time in days, and the vertical axis represented the overall survival (OS) rate.


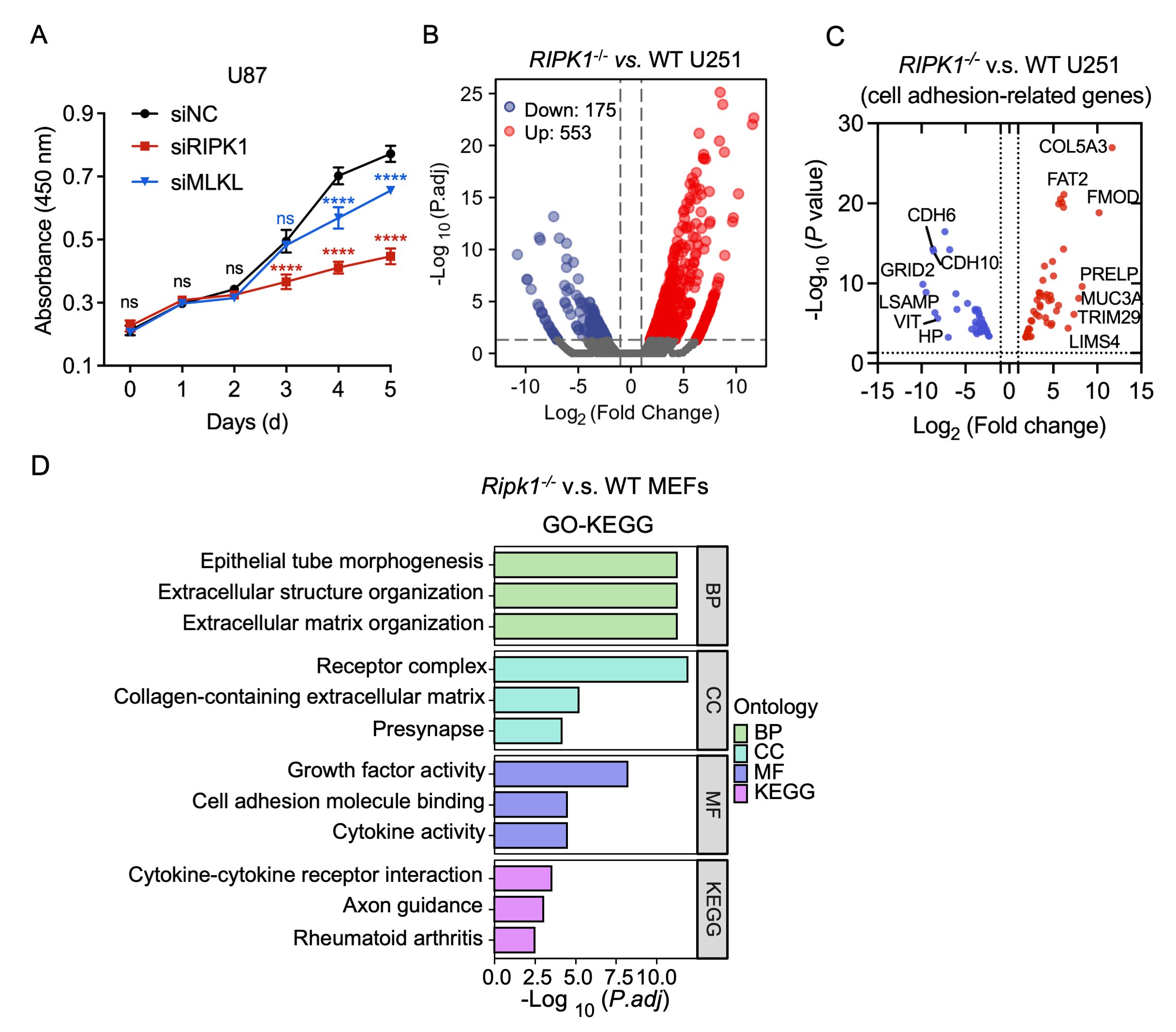


**Figure S6. RIPK1, RIPK3, and MLKL are highly expressed in gliomas and contribute to tumor progression.**(**A**) U87 cells transfected with siRNA were cultured for an additional 5 days, and cell proliferation was assessed using the CCK-8 assay. (**B**) Volcano plot showing differentially expressed genes (DEGs) from RNA-seq analysis comparing RIPK1^–/–^ U251 cells with wild-type U251 cells. Genes with p ≤ 0.05 and fold change ≥ 1.5 or ≤ –1.5 were considered significant. (**C**) Cell adhesion-related genes in **B**. (**D**) GO–KEGG pathway enrichment analysis of Ripk1^–/–^ versus WT MEFs. Data were obtained from the National Omics Data Encyclopedia (OEP002409). All data were shown as means ± SD. **p* < 0.05, ***p* < 0.01, ****p* < 0.001, ns, no significance.
