# Supplementary material for: Necroptotic signaling orchestrates glioblastoma malignancy and potentiates temozolomide response
Source: Cell Death Dis. 2025 Dec 22;16(1):921. doi: 10.1038/s41419-025-08377-3 (PMC12748541; doi:10.1038/s41419-025-08377-3)

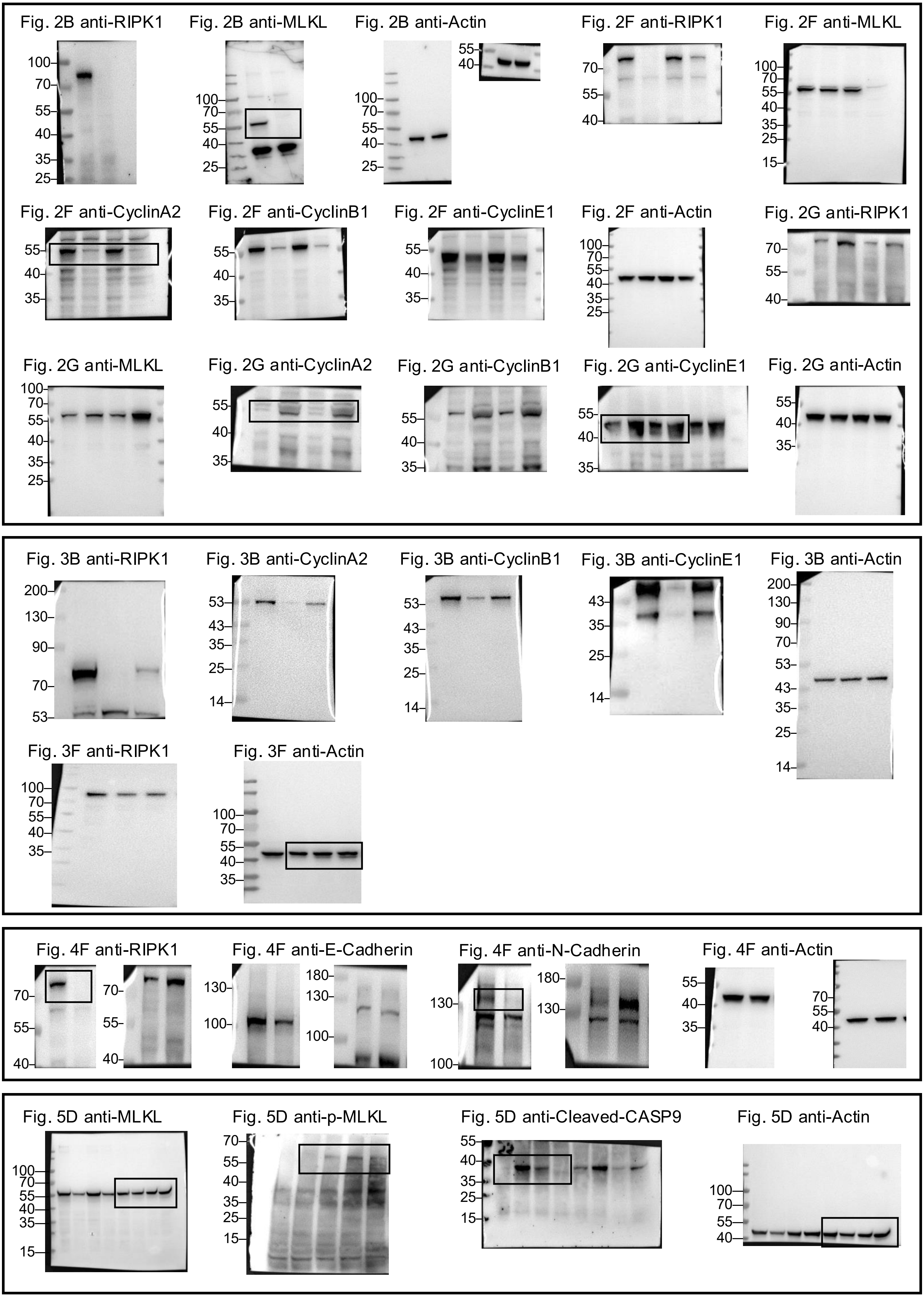
Fig. 2B anti-RIPK1
100 70 55 40 35 25
Fig. 2B anti-MLKL
100 70 55 40 25
Fig. 2B anti-Actin
55 40
100 70 55 40 25
Fig. 2F anti-RIPK1
70 55 40
Fig. 2F anti-MLKL
100 70 55 40 35 25 15
Fig. 2F anti-CyclinA2
55 40 35
Fig. 2F anti-CyclinB1
55 40 35
Fig. 2F anti-CyclinE1
55 40 35
Fig. 2F anti-Actin
100 70 55 40 35 25
Fig. 2G anti-RIPK1
70 55 40
Fig. 2G anti-MLKL
100 70 55 40 35 25
Fig. 2G anti-CyclinA2
55 40 35
Fig. 2G anti-CyclinB1
55 40 35
Fig. 2G anti-CyclinE1
55 40 35
Fig. 2G anti-Actin
55 40 35
Fig. 3B anti-RIPK1
200 130 90 70 53
Fig. 3B anti-CyclinA2
53 43 35 25 14
Fig. 3B anti-CyclinB1
53 43 35 25 14
Fig. 3B anti-CyclinE1
43 35 25 14
Fig. 3B anti-Actin
200 130 90 70 53 43 35 25 14
Fig. 3F anti-RIPK1
100 70 55 40 35
Fig. 3F anti-Actin
100 70 55 40 35
Fig. 4F anti-RIPK1
70 55 40
70 55 40
Fig. 4F anti-E-Cadherin
130 100
180 130 100
Fig. 4F anti-N-Cadherin
130 100
180 130
Fig. 4F anti-Actin
55 40 35
70 55 40
Fig. 5D anti-MLKL
100 70 55 40 35 15
Fig. 5D anti-p-MLKL
70 55 40 35 25 15
Fig. 5D anti-Cleaved-CASP9
55 40 35 25 15
Fig. 5D anti-Actin
100 70 55 40

Fig. S2A anti-RIPK1

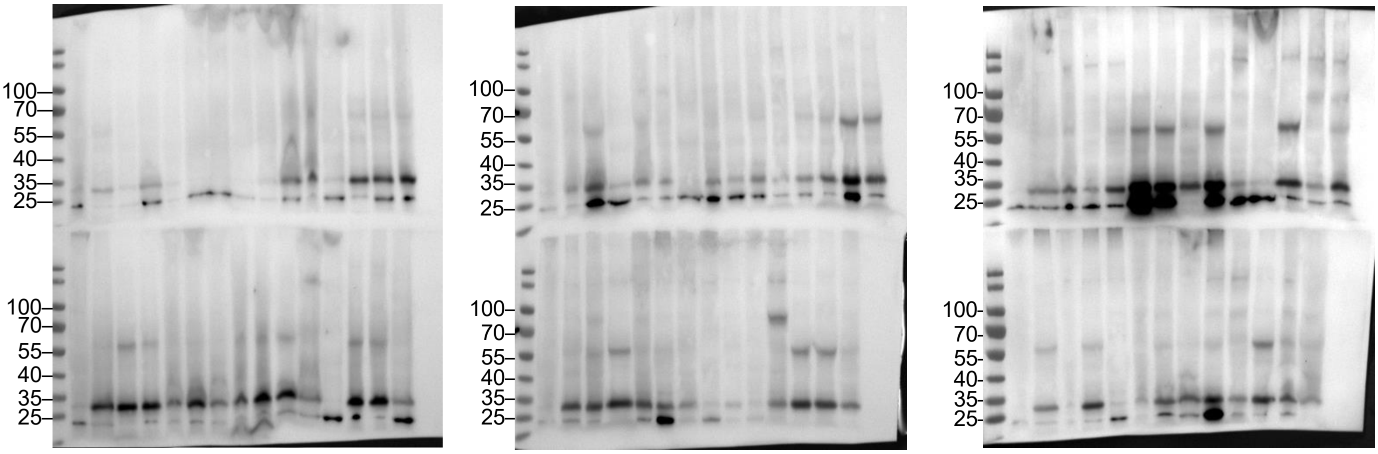

Fig. S2A anti-Actin

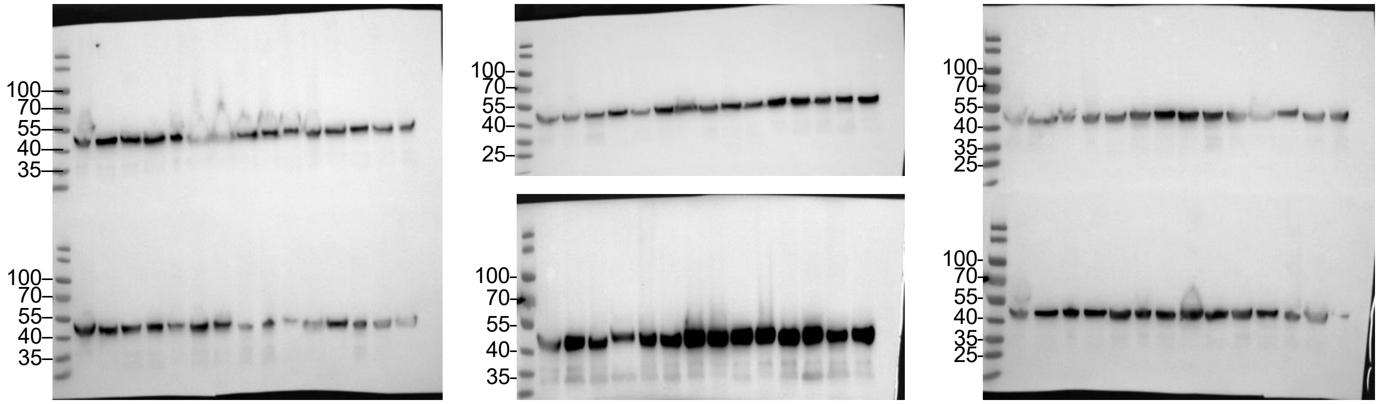

Fig. S2B anti-RIPK3

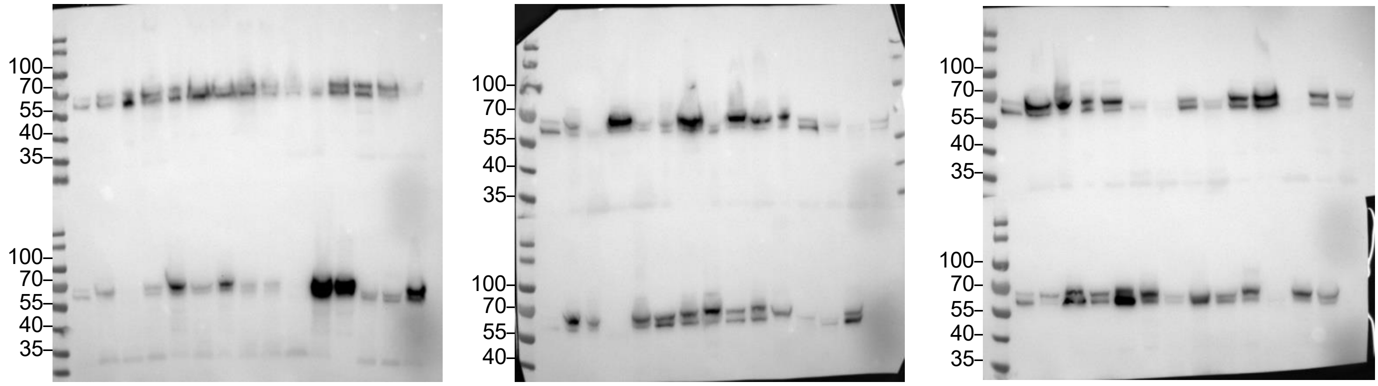

Fig. S2B anti-Actin (RIPK3)

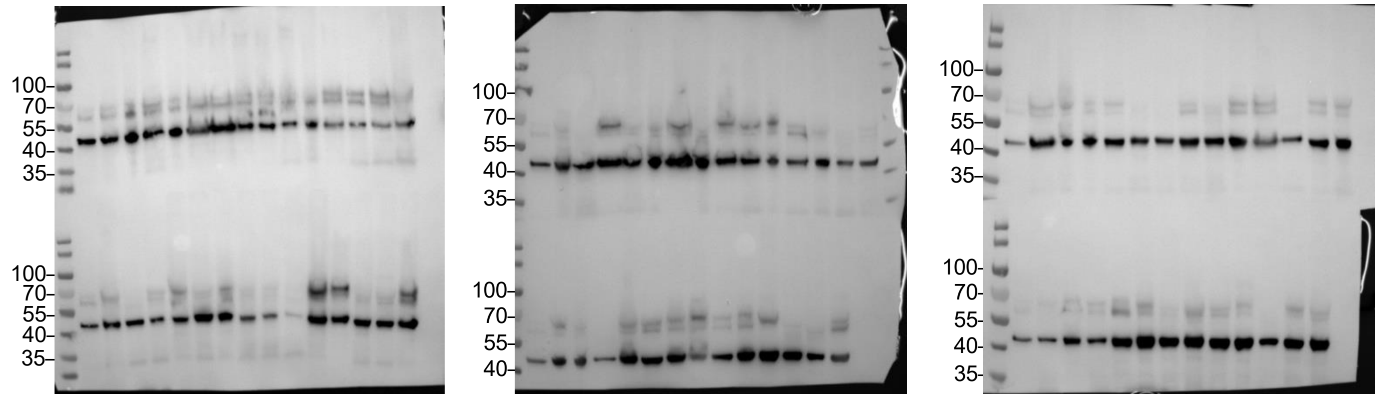

Fig. S2B anti-MLKL

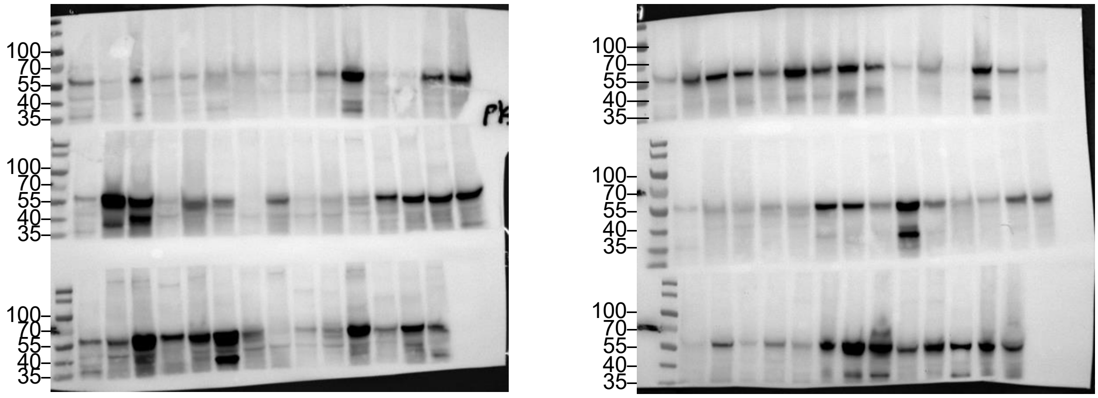

Fig. S2B anti-Actin (MLKL)

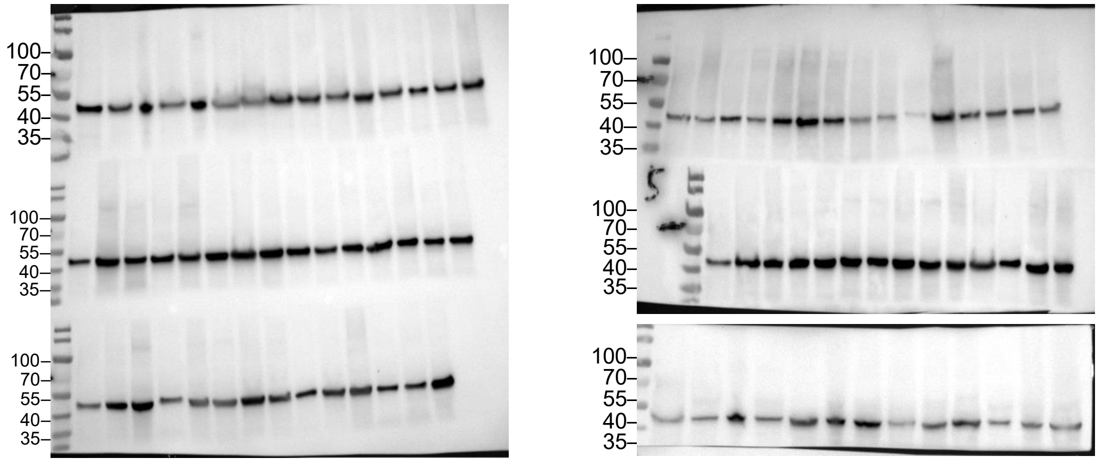

Supplement: Supplementary file 2 — Original Western blots [file 41419_2025_8377_MOESM2_ESM.pdf]
